# Short-Chain Fatty Acid Profiles in Amyotrophic Lateral Sclerosis: Longitudinal Effects of Disease and Mediterranean Diet Intervention

**DOI:** 10.3390/biom15101380

**Published:** 2025-09-28

**Authors:** Anca Moțățăianu, Valentin Ion, Mihai Dumitreasă, Ioana Ormenișan, Lenard Farczadi, Sebastian Andone, Rodica Bălașa, Medeea Maria Roman

**Affiliations:** 1 Department of Neurology, ‘George Emil Palade’ University of Medicine, Pharmacy, Science and Technology of Târgu Mureș, 540142 Târgu Mureș, Romania; anca.motataianu@umfst.ro (A.M.); sebastian.andone@umfst.ro (S.A.); rodica.balasa@umfst.ro (R.B.); 2 1st Neurology Clinic, Mureș County Clinical Emergency Hospital, 540136 Târgu Mureș, Romania; mihai.du96@gmail.com (M.D.); ioana.ormenisan@gmail.com (I.O.); r.medeea@yahoo.com (M.M.R.); 3 Department of Analytical Chemistry and Drug Analysis, Faculty of Pharmacy, ‘George Emil Palade’ University of Medicine, Pharmacy, Science and Technology of Târgu Mureș, 540142 Târgu Mureș, Romania; 4 Drug Testing Laboratory, ‘George Emil Palade’ University of Medicine, Pharmacy, Science and Technology of Târgu Mureș, 540142 Târgu Mureș, Romania; 5 Chromatography and Mass Spectrometry Laboratory, Center for Advanced Medical and Pharmaceutical Research, ‘George Emil Palade’, University of Medicine, Pharmacy, Science, and Technology of Târgu Mureș, 540142 Târgu Mureș, Romania; lenard.farczadi@umfst.ro

**Keywords:** short-chain fatty acids, amyotrophic lateral sclerosis, gut microbiota-brain axis, LC-MS/MS

## Abstract

**Background**: Amyotrophic lateral sclerosis (ALS) evolution is influenced by many dietary factors, biochemical and hormonal inter-relations and gut microbiota. This study focuses on dynamics by conducting a plasmatic quantitative analysis of six of the main short-chain fatty acids (SCFAs) for ALS patients and the shifts in circulating SCFA profiles during ALS progression as well as their potential responsiveness or change due to dietary modulation. **Methods**: A 12-month prospective study in parallel with control group determinations was conducted. The patients diagnosed with ALS were evaluated at the start of the study (T0) followed by a six-month observation time frame (T1) and after another six months of a Mediterranean diet intervention (T2). Plasma SCFAs were determined using liquid chromatography coupled to mass spectrometry to showcase the plasmatic profiles. Correlation between plasma levels of SCFAs and patients’ clinical characteristics next to correlations between plasma SCFA levels at T1 and T2 were performed. **Results**: A significant increase between control group and patients at T0 was observed for acetic, propionic, butyric and hydroxy-butyric acid. Hexanoic acid levels stagnated and 4-methyl-valeric acid concentrations decreased. Evolutions from T1 and T2 impacted acetate, propionate and 4-methyl-valerate. **Conclusions**: The study offers a better understanding regarding the differences in SCFA levels in ALS patients. The Mediterranean diet may impact the levels of acetic and propionic acid, indicating the modulation of SCFA production by gut microbiota.

## 1. Introduction

Amyotrophic lateral sclerosis (ALS), also known as motor neuron disease, is a progressive neurodegenerative disorder characterized by the degeneration of motor neurons in the brain and spinal cord, leading to muscle weakness, paralysis and, finally, to respiratory failure [1]. Despite ongoing research, the pathogenesis of ALS remains incompletely understood, and effective disease-modifying therapies are insufficient [2,3].

Emerging evidence indicates that the gut microbiome may contribute to the heterogeneity observed in ALS, offering important insights into the complex interactions between microbial communities and host physiological processes. Additionally, growing research supports the involvement of systemic metabolic dysfunction, neuroinflammation, and gut microbiota dysbiosis as potential contributors to ALS pathogenesis and progression [4,5,6].

The gut microbiota-brain axis represents a bidirectional communication network between the gut and the central nervous system, via the vagal nerve and systemic immune and metabolic routes, thereby exerting a significant influence on neurological function and health [7]. Short-chain fatty acids (SCFAs), including acetate, propionate, and butyrate, are the main metabolites produced by gut microbial fermentation, primarily derived from dietary fibers in the colon. These metabolites play key roles in modulating immune responses and maintaining intestinal barrier integrity and have an important role in gut-brain crosstalk [8]. Although altered levels of SCFAs have been associated with several neurodegenerative disorders, including Alzheimer’s and Parkinson’s diseases (PD), their role in ALS remains relatively underexplored. However, growing evidence suggests a potential association between dysregulated SCFA profiles and clinical severity in ALS patients [9,10,11].

Epidemiological studies suggest that unhealthy diets, as modifiable lifestyle factors, may increase the risk of neurodegenerative diseases and significantly alter gut microbiota composition and SCFA production [12,13]. The Mediterranean diet, characterized by a high intake of fruits, vegetables, whole grains, legumes, nuts, and olive oil, along with moderate consumption of fish and poultry, has been associated with anti-inflammatory and neuroprotective effects [14]. Notably, this dietary pattern has the potential to enhance the production of short-chain fatty acids (SCFAs) and support gut microbial diversity, representing a promising strategy for nutritional intervention in patients with ALS.

Current evidence indicates that patients with ALS exhibit alterations in gut microbiota composition; however, there are a significant lack of data regarding circulating SCFA levels in this population. Most existing studies have predominantly focused on fecal SCFA concentrations, with insufficient attention given to systemic (plasma) SCFA profiles and their potential associations with clinical severity in ALS. Furthermore, the impact of dietary interventions—particularly anti-inflammatory regimens such as the Mediterranean diet—on circulating SCFA levels and corresponding clinical outcomes in ALS has yet to be thoroughly investigated.

Our primary research question was whether plasma levels of SCFAs can distinguish patients with ALS from healthy controls, and how a Mediterranean diet influences SCFA levels and their association with clinical outcomes in ALS patients.

## 2. Materials and Methods

### 2.1. Study Design and Patients Selection

This longitudinal cohort with a dietary intervention phase single-center study included 44 patients diagnosed with ALS and 40 healthy age- and sex-matched controls. The study was performed in a tertiary care neurology center (Neurology Department of Mures University Clinical Emergency Hospital, Romania). Patients included in this study met the following criteria: (1) definite or probable ALS diagnosis according to the revised El Escorial criteria [15]; (2) confirmed diagnosis of ALS within the previous 12 months; (3) age ≥ 25 years; (4) ability to comply with study procedures; (5) absence of a family history of ALS. Exclusion criteria were (1) a diagnosis of other neurodegenerative disorder; (2) the presence of neurological comorbidities that could potentially interfere with the assessment of ALS progression; (3) comorbidities known to affect gut microbiota or metabolic parameters (i.e., diabetes mellitus, chronic gastrointestinal disease); (4) use of immunosuppressive therapy within the six months preceding study enrollment or antibiotic use within the last three months). All ALS patients received standard ALS therapy (100 mg/day of riluzole) for the entire duration of the study.

ALS patients were longitudinally monitored over a 12-month period in accordance with the study protocol, which included three evaluation time points at six-month intervals—a baseline assessment (T0), a six-month observation period (T1), and a six-month dietary intervention phase (T2). At baseline (T0), 44 ALS patients were evaluated. After the initial six-month observation period (T1), 36 patients were still alive and continued in the study, subsequently initiating the dietary intervention. At the final evaluation (T2, conducted six months after dietary intervention initiation), 30 patients had survived and completed the study. The control group, including 40 participants, was assessed at a single timepoint (at baseline).

All study participants provided informed consent, and the study was approved by the institutional ethics committee.

Ethical approval for the study was obtained from the institutional review boards of both the Mureș County Emergency Clinical Hospital and the “George Emil Palade” University of Medicine, Pharmacy, Science, and Technology of Târgu Mureș (protocol code No. 1841 and date of approval 21 July 2022). The study was conducted in full compliance with relevant ethical guidelines and regulations, in accordance with the principles outlined in the Declaration of Helsinki.

### 2.2. Clinical Assessment

**Figure 1:**
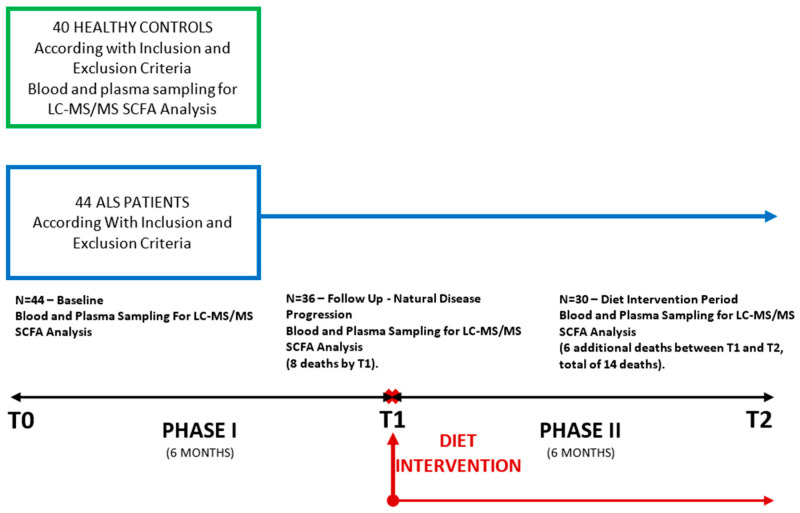
Study flow diagram.

Clinical and paraclinical evaluations were performed at three time points: baseline (T0, at inclusion in the study), after six months of natural disease progression (T1), and after six months of a Mediterranean dietary intervention (T2). The study flow diagram is presented in Figure 1.

At each clinical visit (T0, T1, and T2) all patients underwent comprehensive neurological and neurophysiological assessments to determine disease severity and progression. Neurological functional status was evaluated using the ALS Functional Rating Scale-Revised (ALSFRS-R). The assessment for ALSFRS-R included clinical subscores calculated for bulbar function (ALSFRS-R-B), upper limb function (ALSFRS-R-UL), lower limb function (ALSFRS-R-LL), and respiratory function (ALSFRS-R-R), with a maximum total score of 48 points [16]. All assessments were conducted by an ALS specialist using standardized protocols.

The ALSFRS-R progression rate (ΔPR) was calculated to quantify the rate of functional decline from the time of diagnosis to the study visits. It was determined using the following formula: 48—[(ALSFRS-R at diagnosis—ALSFRS-R at study visit)/duration of symptoms (in months)] [17].

Affective function was assessed using the Beck Depression Inventory II (BDI-II), a validated self-report questionnaire comprising 21 items rated on a four-point scale that quantify the severity of depressive symptoms [18].

### 2.3. Nutritional Intervention

The nutritional intervention for ALS patients, initiated after the T1 visit, consisted of a Mediterranean diet, specifically designed by a specialist in clinical nutrition. Dietary counseling was offered at each visit, and patient adherence was monitored through food records and periodic compliance assessments. The plan followed an approximate macronutrient distribution of 50% carbohydrates (sourced from whole grains, fruits, and vegetables), 30% fats (mainly from plant-based sources such as flaxseed, nuts, seeds, and olive oil, along with small fatty fish rich as herring and sardines), and 20% protein (from lean organic meats, eggs, and fish, excluding red meat). The diet emphasized the inclusion of prebiotic via food intake—such as leeks, garlic, oats, artichokes, and apples—to promote gut microbiome balance. Additionally, it prioritized micronutrient intake, particularly essential fatty acids (alpha-linolenic acid, eicosapentaenoic acid (EPA), docosahexaenoic acid (DHA)), polyphenols, flavonoids, and antioxidant-rich fruits and vegetables (e.g., kale, berries and endives) [19,20].

### 2.4. Plasma SCFA Analysis

#### 2.4.1. Standard Plasma Samples and Real Samples Derivatization Protocol

In order to quantitate the total plasmatic concentrations of six short-chain fatty acids (SCFAs), a derivatization protocol for the carboxylic acids was developed along with a quantitative analytical method based on liquid chromatography (LC) (tandem) mass spectrometry (LC-MS/MS). Standard plasma samples for method development, calibration (CAL), and quality controls (QC) were prepared using pooled plasma obtained from multiple sources of plasma, donated from healthy volunteers. The pooled plasma was used to prepare blank basal endogenic levels of SCFAs, CALs, and QCs levels. For each CAL level or QC level, 2 µL of mix spiking working solutions containing all six SCFAs and 2 µL of mix spiking solution containing three different internal standards (ISTDs) were added to 196 µL of blank plasma. Thus, the obtained targeted concentration intervals for the SCFA were 250–5000 ng/mL (acetic acid-C2-AA and 3-hydroxi butyric acid-C4-OH-3OH-BA each) and 50–1000 ng/mL (propionic acid-C3-PA, butanoic acid-C4-BA, hexanoic acid-C6-HA, and iso-hexanoic acid-iso-C6-4-MVA), respectively. Moreover, employed ISTDs concentrations in plasma were 500 ng/mL (isotope labeled acetic acid-C2-C213), 100 ng/mL (isotope labeled butyric acid-C4-D8), and 100 ng/mL (isotope labeled hexanoic acid-C6-D11), respectively. Each time, a blank plasma level was prepared to evaluate the endogenic levels of SCFAs of blank pooled plasma and to accurately determine the calibration curves for each analysis series. The blank plasma level was prepared starting from 198 µL of blank plasma spiked with 2 µL of mix ISTD spiking solution.

Sample derivatization protocol for spiked BlankISTD, CALs, QCs, and real samples was conducted in parallel. Real samples were one-time thawed at room temperature and gently mixed using a vortex for 30 s, then, 198 µL of each sample was transferred to an Eppendorf 2.0 mL tube. Each sample was spiked with 2 µL of mix ISTD spiking solution and was further gently mixed. Derivatization started with 50 µL of spiked BlankISTD, CALs, and QCs, or 50 µL of spiked real samples was precipitated with 150 µL methanol (MeOH). Further, each sample was vortex-mixed for one min at 2000 rpm using a Velp Scientific Vortex mixer. Next, the samples were centrifuged for five min at 10,000 rpm using an Eppendorf Centrifuge, then 100 µL supernatant was transferred into new 2.0 mL Eppendorf tubes. To the separated supernatant, 50 µL n-(-3-Dimethylaminopropyl)-n’-ehtylcarbodiimde hydrochloride (EDC) 50 mM, 50 µL 3-Nitrophenylhydrazine hydrochloride (3-NPH) 50 mM, and 50 µL Pyridine (Py) 7% (*v*/*v*, in solvent—H_2_O:MeOH = 3:7) were added for derivatization purpose. Under this form, samples were shaken for one hour at the speed of 800 rpm at room temperature, using a Heidolph Multi Reax shaker. After one hour, the derivatization was quenched using 250 µL FA 0.5% (*v*/*v*). The final mixture was homogenized and transferred into an LC glass vial for the LC-MS/MS analysis. Before routine analysis of SCFAs, analytical method performance was assessed using different analytical method validation parameters such as specificity, selectivity, linearity, limit of detection and limit of quantification, accuracy, precision (repeatability and intermediate precision), and system suitability testing.

#### 2.4.2. Liquid Chromatography Mass Spectrometry Analytical Method

The LC-MS/MS platform for the SCFA analysis consisted of a Perkin Elmer Flexar FX-10 UHPLC (ultra-high-performance liquid chromatography) system equipped with a binary pump, a thermostated column compartment, and an autosampler. The UHPLC was hyphenated with a Sciex TripleTOF^®^ 4600 series quadrupole-time of flight mass spectrometer (MS) (Sciex, Darmstadt, Germany). The full separation of the derivatized SCFAs was achieved using a Gemini NX-C18, 100 × 3 mm (3 μm particles size) chromatographic column (Phenomenex, Torrance, California, USA) thermostated at 25 °C. The mobile phase consisted of Solvent A—H_2_O/FA (100/0.2) and Solvent B—Acetonitrile 100%. The flow was set at 0.5 mL/min while the elution was performed under gradient mode. The gradient was started and maintained for 13 min at 30% Solvent B, then Solvent B was linearly ramped to 90% in 17 min. For five min, Solvent B was kept at 90%, and after that, it was swiftly decreased by 30% in order to re-equilibrate the column for five min. Thus, the total analysis time for each injection was 40 min. The injection volume was set at 10 µL; between each injection, a needle wash step involved using 70% H_2_O/FA (100/0.2) and 30% Acetonitrile (100%). The samples were stored in the autosampler at 10 °C.

The MS acquisition parameters were established as follows: The DuoSource TurboV ionization source was set using electrospray ionization mode (ESI) selecting a spray voltage of 4500 V, vaporizer temperature: 500 °C, ion gas source 1: 20 bar, ion gas source 2: 25 bar, curtain gas: 30 bar, declustering potential: −50 V, ion release delay: 39 ms, ion release width: 17. The detection of the analytes was performed using selected reaction monitoring scan mode, while recording the summed signals from multiple transitions, for each derivatized SCFA. Analytical sample preparation and LC-MS/MS analysis variability was corrected using the ISTDs. Peak areas for C2, C3, and C4-OH were corrected with the help of C2-C213 peak areas signal. Peak areas for C4 were corrected with the help of C4-D8 and peak areas for C6 and iso-C6 were corrected using C6-D11. Analyst 1.7 and Multiquant 3.0 Series Sciex software was used for LC-MS/MS control and chromatographic and quantitative data analysis.

### 2.5. Statistical Analysis of SFCAs Longitudinal Study Dynamics

Each study group was assessed for homogeneity of distribution using the Kolmogorov-Smirnov test and for the presence of outliers using Grubbs’ test. Although outliers were identified in some groups, they were not excluded due to the small cohort sizes. Pairwise comparisons for dynamic analyses were conducted using both parametric tests (Student’s t-test with Welch’s correction) and non-parametric tests (Mann-Whitney U test). The dynamics and differences between groups exhibited similar patterns and trends, as demonstrated by both parametric and non-parametric analyses. Statistical analysis was performed using GraphPad Prism version 10.2.3 and Microsoft Excel.

## 3. Results

### 3.1. Clinical and Demographical Characteristics of ALS and Control Groups

Table 1 summarizes demographic and clinical data for control participants and ALS patients across three time points: baseline (T0), after six months of natural disease progression (T1), and after six months of dietary intervention (T2). The number of ALS patients decreased from 44 at T0 to 36 at T1 and 30 at T2, reflecting disease progression and survival. The sex ratio and mean age were comparable across groups and time points. ALSFRS-R scores indicate a general decline in functional abilities, especially in gross motor and total scores, consistent with disease progression. Beck Depression Inventory scores remained relatively stable from T1 to T2, suggesting no major changes in depressive symptoms during the intervention period.

### 3.2. Longitudinal Analysis of Plasma SCFA in ALS Patients and Healthy Controls

We analyzed plasma levels of acetic (AA), propanoic (PA), butyric, 3-OH-butyric (3OH-BA), 4-methyl valeric (4-MVA), and hexanoic acid (HA) in 40 healthy controls (HC) and ALS patients at three time points of the study: at the diagnosis (T0, n = 44), after six months of natural disease progression (T1, n = 36), and after six months of following a Mediterranean diet (T2, n = 30). The results are presented in Table 2.

**Figure 2:**
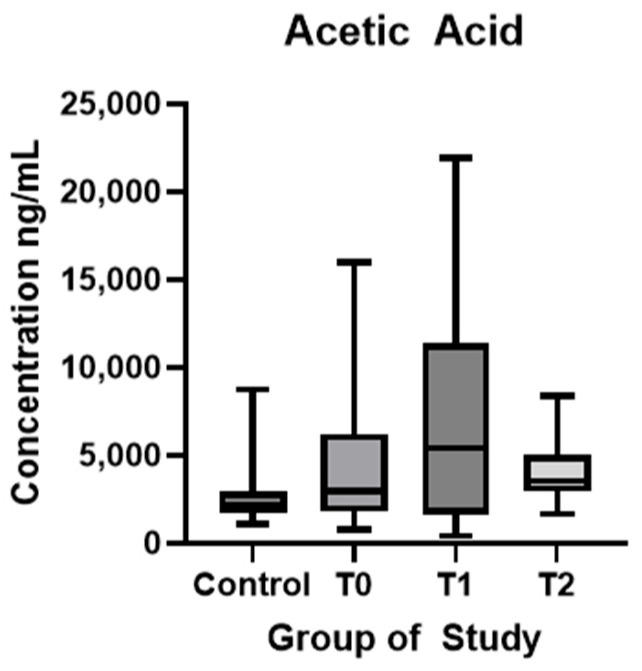
Acetic acid plasma concentration (ng/mL) in controls and ALS patients at T0, T1, and T2. Control—control group, T0—baseline, T1—observation period—six months of natural disease progression, T2—dietary intervention phase—six months of dietary intervention.

In Figure 2, AA showed statistically significantly higher values in ALS patients at the time of diagnosis compared to HC (*p* < 0.01). Statistically significant differences were found between values T1 and T2 (*p* = 0.01).

**Figure 3:**
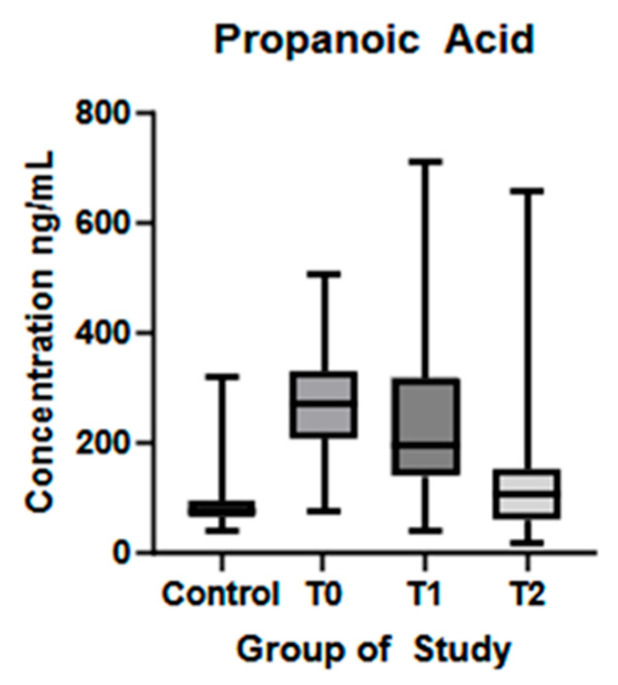
Propanoic acid plasma concentration (ng/mL) in controls and ALS patients at T0, T1, and T2. Control—control group, T0—baseline, T1—observation period—six months of natural disease progression, T2—dietary intervention phase—six months of dietary intervention.

Similar results were found for PA, with a statistically significant difference between values in the HC group and ALS patients at T0 (*p* < 0.01), as seen in Figure 3. Another statistically significant difference was observed between patients at T1 and T2 (*p* < 0.01), with a lower level in T2.

**Figure 4:**
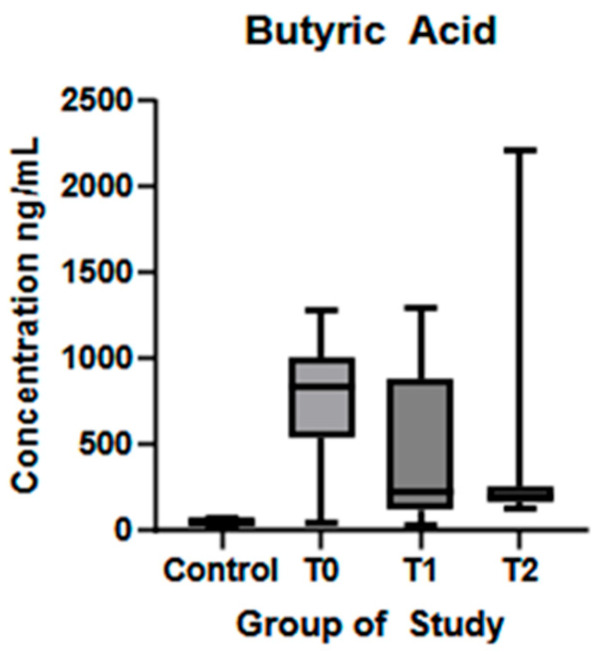
Butyric acid plasma concentration (ng/mL) in controls and ALS patients at T0, T1, and T2. Control—control group, T0—baseline, T1—observation period—six months of natural disease progression, T2—dietary intervention phase—six months of dietary intervention.

BA showed a similar trend, being significantly lower in the control group compared to T0 (*p* < 0.01), and a significant reduction was observed at T1 compared to T0 (*p* = 0.01), represented in Figure 4.

**Figure 5:**
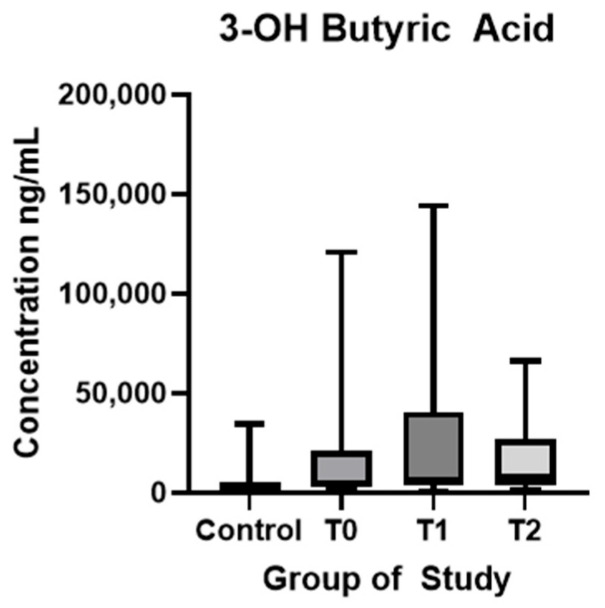
3-hydroxybutyric acid plasma concentration (ng/mL) in controls and ALS patients at T0, T1, and T2. Control—control group, T0—baseline, T1—observation period—six months of natural disease progression, T2—dietary intervention phase—six months of dietary intervention.

3OH-BA showed significantly reduced concentrations in HC compared with T0 (*p* < 0.01). No statistically significant difference was found in the following examinations (Figure 5).

**Figure 6:**
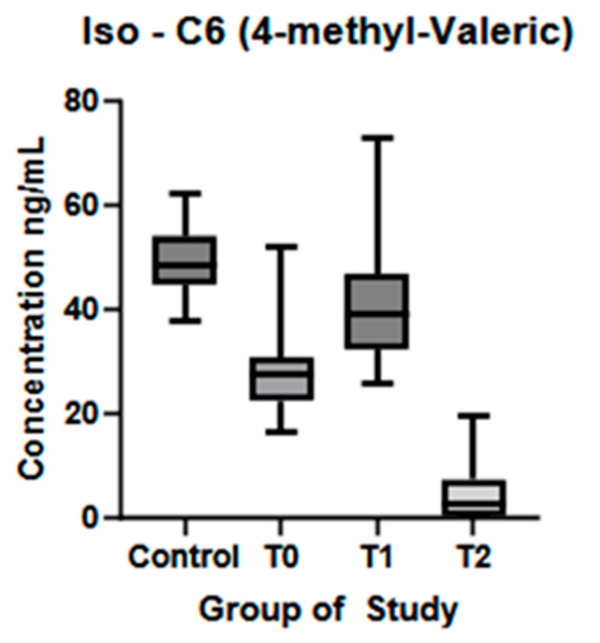
4-methyl-valeric acid plasma concentration (ng/mL) in controls and ALS patients at T0, T1, and T2. Control—control group, T0—baseline, T1—observation period—six months of natural disease progression, T2—dietary intervention phase—six months of dietary intervention.

4-MVA was found in higher concentrations in the control group than at T0 (*p* < 0.0001), with a significant increase in T1 compared to T0 (*p* < 0.01) and a statistically significant reduction in T2 compared to T1 (*p* < 0.01) (Figure 6).

**Figure 7:**
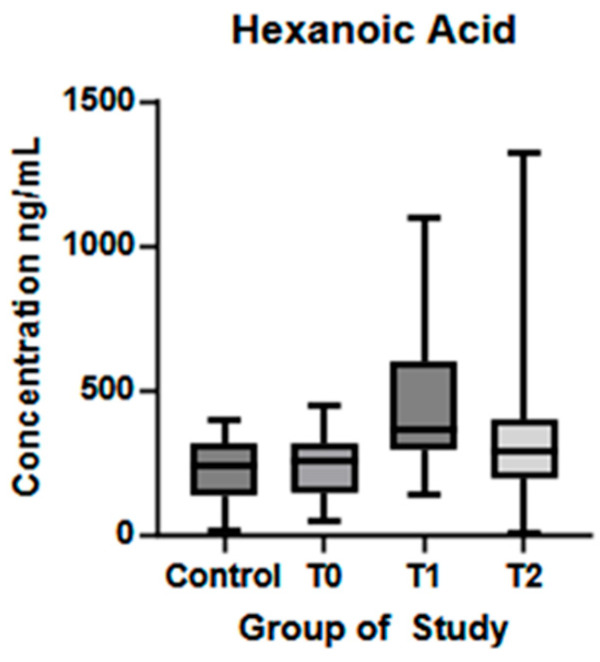
Hexanoic acid plasma concentration (ng/mL) in controls and ALS patients at T0, T1, and T2. Control—control group, T0—baseline, T1—observation period—six months of natural disease progression, T2—dietary intervention phase—six months of dietary intervention.

No significant differences were observed between HA and HC at T0. A statistically significant difference was observed between T0 and T1 (*p* < 0.01), with a reduction of its concentrations in T2, but bearing no statistical significance. HA dynamics are represented in Figure 7.

### 3.3. Correlation Between Plasma Levels of SCFAs and Patients Clinical Characteristics

At T1, statistically significant correlations were observed between AA levels and clinical parameters. A positive correlation was found with the Beck Depression score (*r* = 0.35, *p* = 0.03), while a negative correlation was observed with the ALS-FRS-R fine motor function subscore (*r* = −0.44, *p* < 0.01). BA positively correlated with the ALS-FRS-R fine motor function subscore (*r* = 0.33, *p* = 0.04). 3OH-BA exhibited negative correlations with multiple ALS-FRS-R subscores: respiratory (*r* = −0.33, *p* = 0.04), bulbar (*r* = −0.34, *p* = 0.03), fine motor (*r* = −0.49, *p* < 0.01), and total ALS-FRS-R score (*r* = −0.60, *p* < 0.01).

At T2, no statistically significant correlations were detected between SCFA levels and the monitored clinical parameters.

### 3.4. Correlations Between Expression of SCFAs

At T1, we observed significant correlations among the measured SCFAs: AA positively correlated with 3OH-BA (*r* = 0.68, *p* < 0.01). PA showed positive correlations with BA (*r* = 0.78, *p* < 0.01). 4-MVA and HA were positively correlated (*r* = 0.53, *p* < 0.01).

At T2, these correlations were maintained: AA strongly correlated with 3OH-BA (*r* = 0.80, *p* < 0.01). PA remained positively correlated with BA (*r* = 0.52, *p* < 0.01) and HA (*r* = 0.46, *p* < 0.01). BA and HA showed a positive correlation (*r* = 0.46, *p* < 0.01). All correlations between plasma SCFA levels in ALS patients at T1 and T2 are underlined in Table 3.

## 4. Discussion

In recent years, interest has grown in the relationship between the role of gut microbiota-derived metabolites and neurodegenerative diseases. Although the exact mechanisms remain unclear, SCFAs appear to play a central role, as key metabolic products of the gut microbiota [21,22]. In this study, we examined serum SCFA levels in healthy controls and ALS patients at different stages of the disease and assessed whether a Mediterranean diet could affect these levels and influence disease progression. Notably, four of the SCFAs studied (AA, PA, BA and 3OH-BA) showed increased serum levels at the time of diagnosis compared to healthy controls, while AA, PA, and 4-MVA levels decreased significantly after the six-month dietary intervention. These findings highlight dynamic shifts in circulating SCFA profiles during ALS progression and their potential responsiveness to dietary modulation.

For a better understanding of these findings, it is important to consider the biological characteristics and metabolic roles of these fatty acids. Approximately 95% of SCFAs produced in the large intestine are AA, PA, and BA in a 3:1:1 ratio, while 4-MVA and HA are found in lesser amounts [10,23,24]. Some discrepancies in the literature concern HA, which has six carbon atoms. Most sources classify it as a SCFA [25,26], though some consider it a medium-chain fatty acid (MCFA) [27]. In this study, HA was classified as a MCFA due to its distinct evolution compared to the other SCFAs. 3OH-BA is an important ketone body produced via fatty acid oxidation, mainly by the gut epithelial cells and the liver, serving as an alternative brain energy source during fasting [28,29]. It exerts anti-inflammatory effects by binding to G protein-coupled receptors (GPCRs-GPCR41, GPCR43, and GPCR109A) on various cells—intestinal, nervous, and immune [30,31,32]—while through the inhibition of histone deacetylases (HDACs), SCFAs promote gene expression [33,34,35]. In the CNS, they support brain development, reduce neuroinflammation, modulate neurotransmitters, and aid microglial maturation [36,37].

The gut microbiota-brain axis (GBA) involves bidirectional communication through neural, humoral, and immune pathways, mediated by neuroactive metabolites, including SCFAs [38,39]. All major SCFAs, especially butyrate, cross the BBB via monocarboxylate transporters (MCTs), promoting BBB integrity by restoring the junctional complex proteins, likely via GPR43 signaling [22,30,31,40]. SCFAs also strengthen the intestinal barrier, reduce bacterial translocation, and regulate immune responses by modulating neutrophil activity, suppressing proinflammatory cytokine production, and promoting Treg cell differentiation [11,22,41,42]. Given these neuroprotective and anti-inflammatory effects, it is plausible that changes in SCFA levels may reflect compensatory responses in neurodegenerative conditions such as ALS.

In our cohort, ALS patients at the time of diagnosis showed significantly higher concentrations of the three main SCFAs—acetic, propanoic, and butyric acids—compared to controls. Our data are consistent with other studies which evaluated the plasma concentrations of SCFAs in ALS patients and revealed a significant increase in AA and PA in ALS patients, likely as an adaptive response to meet energy demands [43,44]. Similar findings have been published in a study by Szu-Ju Chen et al. (2022), where they compared fecal and circulating levels of SCFAs in healthy individuals and patients with PD. Their results have shown decreased levels of AA, PA, and BA in feces but higher plasma concentrations, except for AA [45]. In our cohort, significantly higher AA, PA, and BA levels compared to controls may reflect a compensatory response to increased cellular energy demand, possibly via enhanced SCFA production and absorption. This analysis was conducted at ALS diagnosis, relatively early in the disease course. We propose that, similar to microglial activation—where the neuroprotective M2 phenotype dominates the early and slow progression phase—SCFA levels may rise in the initial stages to support neuroregeneration and reduce neuroinflammation [46,47]. An alternative hypothesis points to early gastrointestinal dysfunction preceding clinical onset, which may impair intestinal epithelial integrity and increase gut permeability, allowing abnormal SCFA translocation across the intestinal barrier. Comparative analysis of serum SCFAs levels in neurological diseases, like PD or multiple sclerosis, have shown reduced concentrations in affected individuals [48,49]. These contrasting findings highlight the need for a more detailed investigation into SCFA changes across neurological disorders and disease stages, accounting for treatment and lifestyle factors. After six months of natural disease progression, AA levels increased, while PA and BA levels decreased, but only butyric acid showed a statistically significant change. After six months on a Mediterranean diet, plasma AA and PA levels decreased significantly, while butyric acid levels also declined but without statistical significance. The dietary intervention had little effect on BA, suggesting greater stability of butyrate-producing bacteria in the gut. 3OH-BA showed significant variations between visits. At baseline, ALS patients had higher serum levels than healthy controls, but no significant changes occurred during six months of disease progression. After dietary intervention, 3OH-BA levels decreased without statistical significance. Recent studies highlight its potential neuroprotective role in neurodegenerative diseases like Parkinson’s and Alzheimer’s disease by reducing CNS oxidative stress, increasing blood flow, and upregulating BDNF expression [50,51,52]. 4-MVA was lower in ALS patients at baseline compared to controls, increased significantly at six months, and then decreased significantly after diet. The literature on 4-MVA is limited, and its role in the gut-brain axis remains unclear. One study linked elevated fecal 4-MVA with depression [53], while another found no difference in serum 4-MVA between PD patients and controls or correlation with symptoms [48]. Quantitative analysis of HA showed similar levels in ALS patients and controls at diagnosis but a significant increase after six months of disease progression. Dietary intervention did not significantly affect HA levels. No correlation with clinical parameters was observed at any time point. In a study by Gang Wu et al. (2022), PD patients had lower plasma levels of hexanoic acid compared to healthy controls [49]. Significant changes in SCFA levels after six months of dietary intervention indicate a notable modulation of gut microbial populations in a short time. Among the measured fatty acids, AA, PA, and 4-MVA exhibited significant responses to the diet. The Mediterranean diet, rich in fiber from vegetables, fruits, whole grains, and healthy fats, is known to increase SCFA productions by modulating gut bacterial populations and enhancing fermentation by gut microbiota [14]. Other functional foods, such as curcumin, have been shown to restore SCFA profiles in mice and support neuroprotection by acting as a reactive oxygen species (ROS) scavenger [54,55]. Some studies indicate that short-term dietary changes may not significantly change the gut microbiota or fecal SCFA levels. The gut microbiota’s resilience largely determines the effectiveness of nutritional interventions [56,57]. While a three-month diet change shows little impact on bacterial populations, long-term adherence to a Mediterranean diet increases SCFA production bacteria and fecal SCFA levels [58,59]. According to Szu-Ju Chen et al. (2022), fecal SCFA concentrations may not directly reflect serum levels, highlighting the need to study how gut microbiota modulation affects SCFA absorption and circulation [45]. Moreover, SCFA production varies with fiber type and individual microbiome composition, suggesting nutritional strategies should consider both fiber quality and personal microbiome profiles to optimize outcomes [60].

We believe that the reduced SCFA plasma concentrations in our study group after following the six-month dietary intervention may be related to an improved intestinal barrier and, thus, a reduced absorption of these metabolites in systemic circulation, although limited data are available in the literature regarding plasma levels of SCFAs after dietary intervention. In different studies, following a Mediterranean diet showed increased SCFA production in the intestinal lumen, which lead to an improved gut barrier integrity, reflected by lower lipopolysaccharide-binding protein (LBP) and zonulin concentration and, thus, reduced LBP leakage into the bloodstream [61,62]. As the primary source of energy for the colonocytes, BA consumption may be increased during cell proliferation and differentiation in a healthy gut, while in a pro-inflammatory environment, the intracellular metabolism shifts towards anaerobic glycolysis [63,64]. Also, as AA and PA metabolism is linked to the liver and adherence to a Mediterranean diet has been associated with an improved lipid metabolism, enhancing the liver’s capacity to process SCFAs may result in a reduced circulating concentration of these SCFAs [65,66].

We found significant correlations between SCFA levels and clinical parameters only during the natural progression phase. Acetic acid levels showed a positive correlation with the Beck Depression score, suggesting a potential link between elevated AA and poorer psychological status. Although animal studies, such as those by Weibin Huang et al. (2021), suggest acetate has antidepressant effects through increased histone acetylation (H3, H4), our findings indicate a different trend [67]. Additionally, AA levels negatively correlated with ALSFRS-R fine motor scores, implying that higher acetate may be associated with reduced motor performance. While most research highlights acetate’s neuroprotective and anti-inflammatory effects in animal models—such as reducing neuroglial activation and pro-inflammatory cytokines [68]—our results suggest its role in ALS may differ.

Regarding propanoic acid, no correlations were found between its levels and the clinical aspects of the disease. Numerous studies highlighted its neuroprotective, neuroregenerative, and immunoregulatory effects on both the central and peripheral nervous system, both in human and animal models of PD and ex vivo on Schwan cells and dorsal root ganglia [49,69,70].

The positive correlation between BA and ALSFRS-r fine motor SCORES suggests that higher BA levels may be linked to better motor function in ALS patients. Known for its neuroprotective and anti-inflammatory effects, BA is the most potent HDAC inhibitor among SCFAs, with ≈80% efficiency, followed by PA [21,71,72]. In Alzheimer’s models, BA improves learning and memory by enhancing gene expression and histone acetylation [73]. In ALS mouse models, early gut dysbiosis was linked to reduced butyrate-producing bacteria and impaired intestinal barriers [74]. In mouse models of Parkinson’s disease and brain injury, sodium butyrate increased occludin and zonula occludens-1 (ZO-1) expression in the hippocampus and frontal cortex, supporting BBB function [75,76], while supplementation with 2% butyrate in G93A mice restored gut integrity and extended lifespan [77]. Human PD patients showed lower fecal BA levels, which correlated with poorer motor and cognitive performance and more severe depressive symptoms, while a case-control study on patients with major depressive disorder revealed that higher plasma concentrations of butyrate were associated with a greater likelihood of remission [45,78]. Negative correlations between 3OH-butyric acid and ALSFRS-R respiratory, bulbar, fine motor, and total score suggest that higher levels of 3OH-BA are associated with poorer overall function. This contrasts with the existing literature, where 3OH-BA has been shown to inhibit histone deacetylases, reduce oxidative stress and inflammation, and protect against dopaminergic neurodegeneration in mouse models [29]. Xiao-Qiang et al. (2007) reported reduced apoptosis in mouse glial cells following 3OH-BA treatment [28]. In subsequent evaluations—during disease progression and after dietary intervention—no significant correlations were found between SCFA levels and clinical outcomes.

This study has several limitations. The number of ALS patients included was limited, which may reduce the statistical power of the findings; however, studies on animal models might not adequately reflect the complexity of ALS in humans, highlighting the value of even small-scale human studies. Additionally, microbiota sequencing could improve our findings by directly linking the SCFA changes to microbial shifts. Recent studies have shown variations in the microbiomes of ALS patients, including between different subtypes of the disease, while SCFA levels remained stable [79]. Lastly, a six-month dietary intervention might not be sufficient to capture long-term changes in SCFA levels or sustained clinical effects, emphasizing the need for extended periods of monitoring.

## 5. Conclusions

This longitudinal study reveals significant alterations in plasma SCFA levels in ALS patients, both during natural disease progression and after dietary intervention. At diagnosis, patients showed elevated levels of acetic, propanoic, butyric, and 3-hydroxybutyric acids compared to healthy controls, with dynamic shifts over time. The Mediterranean diet led to reduced acetic and propanoic acid levels, indicating the modulation of SCFA production by gut microbiota, while butyric and hexanoic acids were less affected—possibly reflecting compensatory metabolic or anti-inflammatory responses. These findings demonstrate that plasma SCFA profiles can distinguish ALS patients from controls and that a Mediterranean diet may modulate SCFA levels. Correlations between SCFA levels and clinical features, including motor function and depressive symptoms, suggest a potential role of gut-derived metabolites in ALS pathophysiology. These findings support the view that microbial and metabolic factors may influence disease progression and that dietary strategies could offer a viable supportive therapy.

## Figures and Tables

**Table 1:** Demographic and clinical characteristics of ALS patients and healthy controls across study time points. Legend: Control—control group, T0—baseline, T1—observation period—six months of natural disease progression, T2—dietary intervention phase—six months of dietary intervention.

		Control	ALS		
			T0	T1	T2
Patients number		40	44	36	30
Sex ratio (M:F)		24:16	28:16	23:13	20:10
Age (mean ± SD) (years)		56.97 ± 11.83	58.05 ± 12.14	58.17 ± 11.78	57.43 ± 12.39
ΔPR		NA	0.92 ± 1.05	0.75 ± 0.67	0.54 ± 0.34
ALS-FRS-R subscore	Respiratory	NA	10.95 ± 1.61	11.00 ± 1.33	10.23 ± 2.34
	Bulbar	NA	9.64 ± 2.63	9.39 ± 2.71	9.27 ± 2.88
	Gross motor	NA	6.42 ± 3.23	8.00 ± 2.82	5.57 ± 3.14
	Fine motor	NA	7.24 ± 3.42	6.53 ± 3.39	6.23 ± 3.50
	Total	NA	33.95 ± 7.92	32.69 ± 7.00	31.58 ± 8.91
Beck Depression score		NA	NA	13.69 ± 8.17	13.17 ± 9.65

**Table 2:** Plasma short-chain fatty acid concentrations in ALS patients and controls at T0, T1, and T2 with statistical comparisons. Legend: Control—control group, T0—baseline, T1—observation period—six months of natural disease progression, T2—dietary intervention phase—six months of dietary intervention.

SCFA	Control	ALS					
		T0	T1	T2	C vs. T0	T0 vs. T1	T1 vs. T2
Acetic acid (ng/mL) (mean ± SD)	2622 ± 1724	4633 ± 3948	7057 ± 6445	4223 ± 1757	*p* < 0.01 *	*p* = 0.05	*p* = 0.01 *
Propanoic acid (ng/mL) (mean ± SD)	85.11 ± 44.08	269.9 ± 105.7	231.9 ± 137.9	154.4 ± 169.5	*p* < 0.01 *	*p* = 0.17	*p* = 0.04 *
Butyric acid (ng/mL) (mean ± SD)	51.55 ± 12.44	743.8 ± 367	519.6 ± 429.3	367.4 ± 505.3	*p* < 0.01 *	*p* = 0.01 *	*p* = 0.19
3-OH-butyric acid (ng/mL) (mean ± SD)	5635 ± 7672	15,850 ± 23,738	24,049 ± 31,527	15,429 ± 15,525	*p* < 0.01 *	*p* = 0.20	*p* = 0.15
4-methyl-valeric acid (ng/mL) (mean ± SD)	49.32 ± 6.30	20.15 ± 8.42	40.67 ± 10.80	4.55 ± 5.11	*p* < 0.01 *	*p* < 0.01 *	*p* < 0.01 *
Hexanoic acid (ng/mL) (mean ± SD)	219.7 ± 112.3	242.3 ± 112	449.1 ± 255.7	382.1 ± 297.3	*p* = 0.35	*p* < 0.01 *	*p* = 0.33

* Statistical significant difference.

**Table 3:** Correlations between plasma SCFA levels in ALS patients at T1 and T2. T1—observation period—six months of natural disease progression, T2—dietary intervention phase—six months of dietary intervention.

SCFA		T1	T2
Acetic acid	3-OH butyric acid	*r* = 0.68, *p* < 001	*r* = 0.80, *p* < 0.01
Propanoic acid	Butyric acid	*r* = 0.78, *p* < 0.01	*r* = 0.52, *p* < 0.01
4-methyl-valeric acid	Hexanoic acid	*r* = 0.53, *p* < 0.01	*r* = 0.44, *p* = 0.01
3-OH butyric acid	Hexanoic acid	*r* = −0.42, *p* < 0.01	*r* = 0.07, *p* = 0.71
Propanoic acid	Hexanoic acid	*r* = −0.03, *p* = 0.82	*r* = 0.46, *p* < 0.01
Butyric acid	Hexanoic acid	*r* = −0.03, *p* = 0.83	*r* = 0.46, *p* = 0.01

## Data Availability

The data presented in this study are available only by request but will not be publicly available due to restrictions from our funding contract.

## Abbreviations

The following abbreviations are used in this manuscript:

3-NPH	3-Nitrophenylhydrazine Hydrochloride
3OH-BA	3-Hydroxybutyric Acid
4-MVA	4-Methyl-Valeric Acid
AA	Acetic Acid
ALS	Amyotrophic Lateral Sclerosis
BA	Butyric Acid
BBB	Blood-Brain Barrier
CAL	Calibration
CNS	Central Nervous System
DHA	Docosahexaenoic Acid
EDC	N-(-3-Dimethylaminopropyl)-N’-Ehtylcarbodiimde Hydrochloride
EPA	Eicosapentaenoic Acid
ESI	Electrospray Ionization Mode
FA	Formic Acid
GBA	Gut Microbiota-Brain Axis
GPCR(s)	G Protein-Coupled Receptor(S)
HA	Hexanoic Acid
HC	Healthy Control
HDAC(s)	Histone Deacetylase(S)
ISTDs	Internal Standards—Isotope Labeled
LBP	Lower Lipopolysaccharide-Binding Protein
LC-MS/MS	Liquid Chromatography (LC) (Tandem) Mass Spectrometry
MCFA	Medium-Chain Fatty Acid
MCTs	Monocarboxylate Transporters
MeOH	Methanol
MS	Mass Spectrometer
PA	Propanoic Acid
PD	Parkinson’s Disease
Py	Pyridine
QC	Quality Controls
SCFAs	Short-Chain Fatty Acids
T0	Baseline
T1	Observation Period—Six Months of Natural Disease Progression
T2	Dietary Intervention Phase—Six Months of Dietary Intervention
UHPLC	Ultra-High-Performance Liquid Chromatography
